# Comparative Evaluation of Light‐Driven Catalysis: A Framework for Standardized Reporting of Data**

**DOI:** 10.1002/anie.202114106

**Published:** 2022-06-13

**Authors:** Dirk Ziegenbalg, Andrea Pannwitz, Sven Rau, Benjamin Dietzek‐Ivanšić, Carsten Streb

**Affiliations:** ^1^ Institute of Chemical Engineering Ulm University Albert-Einstein-Allee 11 89081 Ulm Germany; ^2^ Institute of Inorganic Chemistry I Ulm University Albert-Einstein-Allee 11 89081 Ulm Germany; ^3^ Institute of Physical Chemistry and Center of Energy and Environmental Chemistry Jena (CEEC Jena) Friedrich Schiller University Jena Helmholtzweg 4 07743 Jena Germany; ^4^ Department Functional Interfaces Leibniz Institute of Photonic Technology Jena (IPHT) Albert-Einstein-Straße 9 07745 Jena Germany; ^5^ Department of Chemistry Johannes Gutenberg University Mainz Duesbergweg 10-14 55128 Mainz Germany

**Keywords:** Comparability, Heterogeneous, Homogeneous, Light-Driven Catalysis, Photocatalysis

## Abstract

Light‐driven homogeneous and heterogeneous catalysis require a complex interplay between light absorption, charge separation, charge transfer, and catalytic turnover. Optical and irradiation parameters as well as reaction engineering aspects play major roles in controlling catalytic performance. This multitude of factors makes it difficult to objectively compare light‐driven catalysts and provide an unbiased performance assessment. This Scientific Perspective highlights the importance of collecting and reporting experimental data in homogeneous and heterogeneous light‐driven catalysis. A critical analysis of the benefits and limitations of the commonly used experimental indicators is provided. Data collection and reporting according to FAIR principles is discussed in the context of future automated data analysis. The authors propose a minimum dataset as a basis for unified collecting and reporting of experimental data in homogeneous and heterogeneous light‐driven catalysis. The community is encouraged to support the future development of this parameter list through an open online repository.

## Graphical abstract

This Scientific Perspective describes current challenges in determining, reporting, and comparing performance indicators in homogeneous and heterogeneous light‐driven catalysis. The need for comparative data analysis in the field is highlighted, and its role in future data reporting and big‐data analysis is discussed. A minimum set of experimental parameters to be reported for light‐driven catalysis is proposed.

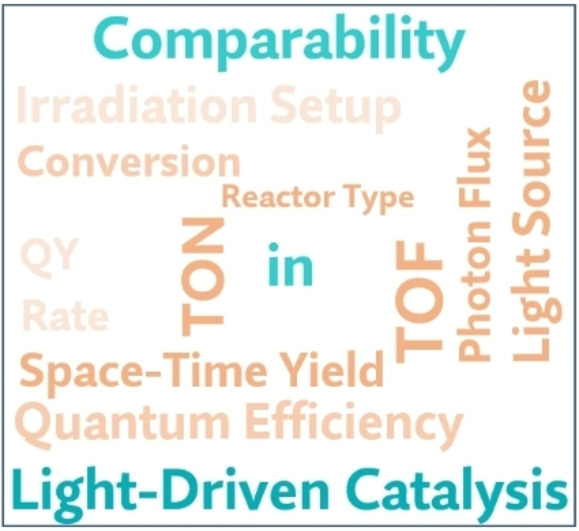

## 1 Introduction

### 1.1 General Background

Visible light‐driven chemistry has attracted widespread interest from academia and industry as it enables carbon‐neutral energy conversion and storage. Central pillars are photo(electro)chemical water splitting,[^1^, ^2^] the activation of inert molecules such as CO_2_ ^[3]^ and N_2_,[^4^, ^5^] the use of (visible) light for organic photochemical conversions,[^6^, ^7^, ^8^, ^9^] organic photoredox catalysis,[^10^, ^11^, ^12^] and synthetic fuel synthesis.^[13]^ Efforts worldwide have been focused on developing sustainable and technologically viable components for light‐driven catalysis, including light‐absorbers, redox‐mediators, and catalysts.[^14^, ^15^, ^16^] In addition, advanced photoreactors are developed to maximize photon harvesting and utilization.[^17^, ^18^, ^19^] This has led to pioneering research in homogeneous and heterogeneous light‐driven catalysis, highlighting the vast possibilities for energy technologies and industrial chemistry.^[20]^

However, to further develop light‐driven catalytic systems, it is imperative to experimentally identify, report, and compare reactivity, stability, and performance. Thus, well‐defined protocols, standardized experimental setups, and experimentally accessible performance indicators are urgently required to provide quantitative comparability[^21^, ^22^, ^23^] and unbiased, reliable, and reproducible performance evaluation across multiple systems and laboratories.[^22^, ^24^, ^25^] A prime example of the vast benefits of quantitative comparability in a research field is seen in organic photovoltaics research, where stability^[26]^ and efficiency^[27]^ reporting has been unified, based on consensus within the research community. This has led to stringent and transparent protocols for the reporting of organic photovoltaics.^[28]^

**Figure 1:**
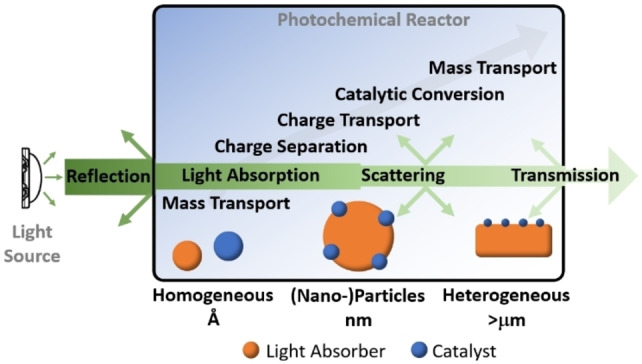
Typical experimental setup, principal steps, and reactive component types employed in light‐driven catalysis.^[29]^

In contrast, light‐driven catalysis comprises an enormous range of systems where mechanistic steps and effects span across many orders of magnitude in time and space (Figure 1).[^29^, ^30^] The complex interplay between system components, reaction conditions, and community‐specific reporting strategies has thus far prevented the development of unified comparability protocols by the communities. However, research organizations across the globe have expressed the need for open and transparent data reporting, which has led to the so‐called FAIR principles (findable, accessible, interoperable, reusable), which will form the basis for algorithm‐based, automated data analyses.[^31^, ^32^] Thus, there is an urgent need for comparable and reliable and open data recording and reporting in light‐driven catalysis. This will allow researchers to identify performance limitations to focus on areas with high development potential, and to use existing, published information for knowledge‐based materials and reaction development.

### 1.2 Challenges in Determining, Evaluating, and Comparing Light‐driven Catalytic Performance

Light‐driven catalysis typically relies on the interplay between multiple components and processes (Figure 1). The initial step depends on a light‐absorber that uses photon energy to drive charge separation. If electron–hole recombination can be sufficiently suppressed, the desired redox equivalent (i.e., the electron (e^−^) or the electron hole (h^+^)) is transferred to the respective reaction site, which drives the catalytic turnover. Often, sacrificial electron donors or acceptors are used to transfer redox equivalents.[^33^, ^34^] (Photo)reaction engineering and reactor design are critical to optimize light input and mass transfer, particularly in multiphase systems. However, they are often neglected in fundamental light‐driven catalysis research, where the focus is on the performance of molecular components.^[29]^

As catalytic processes typically show maximum performance in a narrow window of operation, defining a standard set of reaction conditions would be inherently biased and is not used in the field. Instead, a set of experimentally accessible, bias‐free performance indicators is required, which can be widely used to evaluate and compare light‐driven catalytic performance. While this Scientific Perspective outlines the steps required for objective comparison in light‐driven catalysis, researchers have to evaluate whether comparison between two systems is meaningful. The authors believe that comparison is most valuable for systems performing related catalytic reactions, while comparison between two unrelated systems is not trivial and often also not necessary.

## 2 Chemical Parameters Affecting Reactivity in Light‐Driven Catalysis

### 2.1 General Considerations Regarding Reaction Conditions

**Figure 2:**
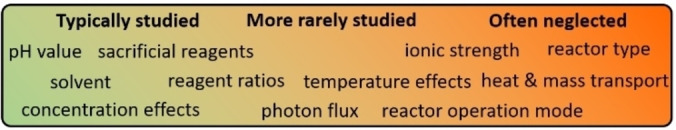
Typical experimental parameters in light‐driven catalysis and the frequency of their study in light‐driven catalysis.

Light‐driven catalysis is controlled by the species that directly participate in the reaction (e.g., light absorber, catalyst, sacrificial electron donors/acceptors, reagents, intermediates, and products; i.e., the reaction system).^[11]^ However, other seemingly non‐participating components, such as solvents,[^35^, ^36^] counter‐ions,^[37]^ buffers,^[38]^ or dissolved gases^[39]^ can have unexpected effects that alter reactivity dramatically and therefore also require careful study.[^40^, ^41^, ^42^] For a summary of key reaction parameters, see Figure 2.

**Concentration and ratio of reagents** significantly affect photochemical reactivity and can lead to changes from virtually non‐reactive to highly reactive.[^43^, ^44^] Often the exact interplay between components is not understood as little is known about the underlying reaction mechanisms.[^37^, ^45^, ^46^]

**The electron donor/acceptor** affects the observed reactivity, as redox potentials (and their dependence on protonation degree; i.e., solution pH)^[47]^ as well as interactions with other components^[48]^ control light‐driven catalytic performance.[^49^, ^50^, ^51^]

**Solvent type and solution pH** influence solubilities, component stability, and component interactions. The solvent itself can (and regularly does) participate in reactions, either by providing a dielectric, polarizable medium for the reaction,^[52]^ or by intermolecular interactions (e.g., coordination) to reactive species.^[35]^ Solvents can even act as electron donors or acceptors and thereby interfere with the photoredox processes.[^52^, ^53^]

**Temperature and pressure** of the reaction system need to be monitored and documented, in particular for biphasic gas–liquid reactions, as changes in temperature^[54]^ and pressure^[55]^ can have a major impact on photochemical reactivity.

### 2.2 Specific Challenges in Homogeneous Light‐Driven Catalysis

Light‐driven reactions in solution are highly dependent on the kinetics of many elementary processes, which need to occur in a specific order (Figure 1). Key factors to be considered are:

**Intermolecular and supramolecular interactions**, including electrostatic, van der Waals, π‐π stacking or hydrogen‐bonding interactions, as well as host–guest complex formation can control the aggregation behavior of molecular components in solution.[^56^, ^57^] This can lead to component pre‐aggregation (e.g., ion pairing), which is beneficial for fast charge transfer.^[58]^ However, it can also result in colloid formation, precipitation,^[37]^ and fast charge recombination,^[59]^ which limits reactivity by reducing the amount of active components in solution. Note that these effects frequently lead to changes of the optical density of the reaction solution.

**Kinetic rate matching**. The elementary steps in light‐driven catalysis occur on vastly different time scales, ranging from femto‐ to milliseconds, and beyond.^[60]^ For efficient catalysis, these time scales need to be aligned by tuning of the reaction conditions.[^54^, ^61^] For example, diffusion‐controlled processes such as oxidative or reductive quenching of a photo‐excited light‐absorber by a diffusing electron donor/acceptor have a higher probability of occurring when the light absorber features long excited state lifetimes.^[62]^ Excited state lifetimes, however, are themselves affected by the reaction conditions (e.g., solvent polarity),^[63]^ similar to diffusion‐controlled processes, which are strongly affected; e.g., by temperature, reagent concentration, and solvent viscosity.[^54^, ^64^]

### 2.3 Specific Challenges in Heterogeneous Light‐Driven Catalysis

Heterogeneous light‐driven catalysis uses solid‐state compounds as a light absorber and catalyst, or as support for immobilizing molecular components.^[65]^ In both cases, the following factors affect catalytic performance:

**Optical effects**, such as scattering and reflection at the interface between the solvent and the solid‐state compounds, affect the photon flux at catalyst particle, and thus the overall reaction rate.[^66^, ^67^, ^68^]

**Mass transport** affects catalytic reactivity by controlling convective and diffusive transport of reactants to and products away from the catalyst or light absorber,[^69^, ^70^] and by convective mass transport in the bulk phase.^[64]^ Moreover, reagent adsorption and product desorption at the particle surface are key processes to be considered. Concentration gradients, which form during catalysis, can affect the overall performance.^[71]^

## 3 Technical Parameters Affecting Catalytic Activity

### 3.1 Challenges in Characterizing the Experimental Irradiation Setup ‐ from Light Source to Reactor

The optical characteristics of the light source (i.e., emitted photon flux, emission wavelengths, and emission geometry) are key parameters for light‐driven catalysis.[^17^, ^54^] However, reporting of these parameters is still not standardized and can lead to challenges in understanding and reproducing published experimental data.

**Figure 3:**
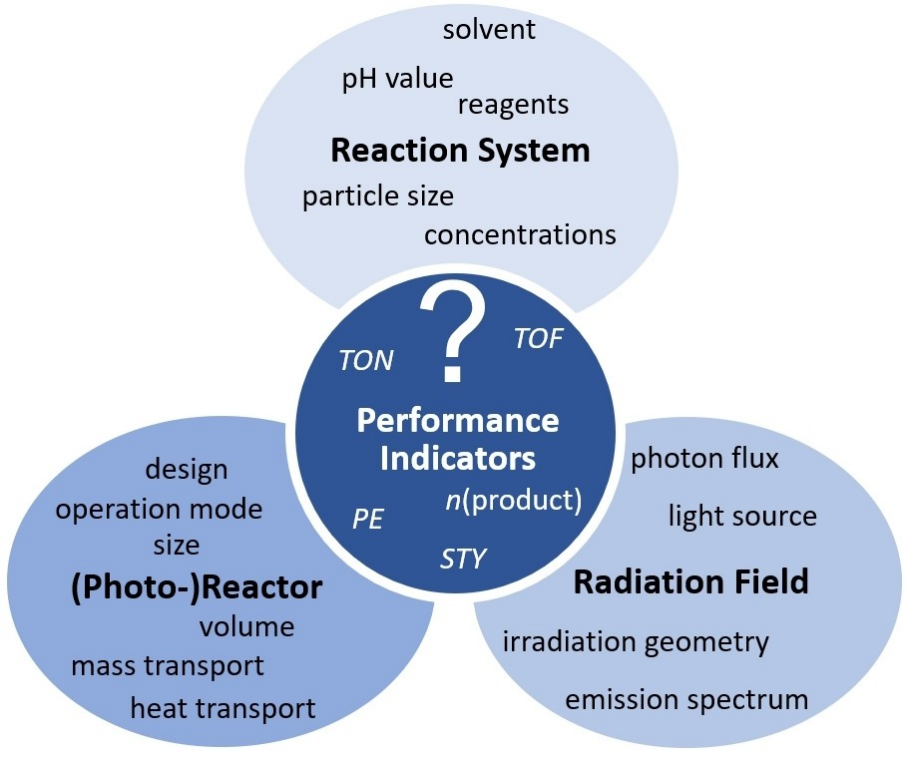
Illustration of the key parameters affecting light‐driven catalysis and the challenge of determining unbiased performance indicators.

**Spectrally resolved incident photon flux** describes the photon flux that reaches the inside of the reactor. It is an ideal basis for quantitative interpretation of light‐driven catalytic reactivity data, as incident photons can be considered additional reagents required for the light‐driven process under study.[^72^, ^73^] The incident photon flux depends on the light source, the beam path, the irradiation geometry, and the reactor wall materials (Figure 3).[^74^, ^75^] A detailed description of the experimental setup is essential to ensure reproducibility and comparability. The incident photon flux can be experimentally determined by actinometry or similar methods.^[76]^ Note that actinometry is time‐consuming, experimentally tedious, and has to be performed for each individual reaction vessel and experimental setup.[^77^, ^78^, ^79^, ^80^]

As a practical alternative to improve evaluation and reproducibility, we recommend the following minimum information to be reported: the geometric arrangement and relative positioning of light source and photoreactor, as well as the emission characteristics of the light source, and the overall reactor design and the reactor materials.[^81^, ^82^] The use of well‐characterized or standardized reactor setups represents another approach to ensure reproducibility.[^83^, ^84^, ^85^, ^86^, ^87^] Furthermore, light‐source aging needs to be considered; for instance, the intensity of mercury vapor lamps drops sharply during the first 500 hours and typically reaches 70 % of the initial intensity after 2000 hours of operations. In contrast, LEDs show a linear decrease of the emitted intensity and reach 70 % of the initial intensity after 20 000 hours.^[75]^ Note that handheld optical power meters are ideal to measure and document the (integral) power output of a light source before starting an experiment.

**The absorbed photon flux** forms a basis for photonic efficiency determinations, and therefore requires quantitative determination (see below). However, determining photon fluxes is error prone and requires careful experimentation.[^77^, ^78^, ^88^] This can be addressed by establishing a thorough understanding of the radiation field around a photoreactor by multidimensional radiometric measurements combined with numerical methods; e.g., ray tracing.^[89]^

**The photoreactor design** affects the photon flux as well as the local reaction environment. Hydrodynamic parameters such as (non‐)ideal mixing, dead zones, and residence time distributions have significant impacts on light‐driven reactivity.^[90]^

### 3.2 Reaction Engineering Challenges

Interfacing molecular catalysis with reaction engineering holds vast possibilities to increase photochemical performance.^[91]^ A lack of knowledge at this interface is a major obstacle for scale‐up and industrial deployment of light‐driven reactions.[^92^, ^93^, ^94^]

**Temporal and spatial changes of reaction conditions**. Light‐intensity drops exponentially along the ray trajectory (Beer–Lambert law). For strongly absorbing systems, or systems using high‐intensity irradiation, this results in pronounced reaction rate gradients along the light‐beam trajectories. For example, a highly absorbing reaction system with an overall absorbance *A*=2 will show a 50 % decrease in light intensity within only 15 % of the optical beam path in the reactor. Large reactor volumes are therefore not irradiated and cannot participate in light‐driven reactions. The problem is compounded if mixing within the reactor is not ideal, which can result in detrimental side reactions; e.g., increased degradation of light absorber or catalyst.^[95]^

Reagent consumption and intermediate/product formation further affect light‐driven catalysis; their detailed impact on reactivity is often not well understood. Possible effects range from time‐dependent changes of the optical density of the reaction solution to aggregation‐induced reactivity,[^96^, ^97^] positive/negative feedback loops,^[98]^ product inhibition[^48^, ^99^] and effects of dynamic irradiation conditions.[^90^, ^100^] Based on well‐documented reporting of the relevant experimental parameters, these phenomena can be understood and used to develop improved reaction systems.

**Impact of reactor design and operation**. Continuously and discontinuously operated reactors feature strikingly different properties. Ideal, discontinuous batch reactors (on the lab‐scale, this corresponds to a stirred flask) have a spatially homogeneous concentration field, which changes with the reaction progress. In contrast, ideal, continuous plug‐flow reactors (on the lab‐scale, this corresponds to a capillary reactor) show a temporally constant but spatially changing concentration field along the direction of flow. The extent that reactants are mixed with products differs, leading to a different availability of reactants and products near the catalytic sites during the reaction progress and thus to a different reaction performance. Differences in heat and mass transport depend on the reactor type, and all these factors directly affect the (light‐driven) reactivity. Various laboratory scale reactors have recently been reported to address the challenges of comparability.[^85^, ^87^, ^101^, ^102^] Specific chemical challenges, such as enhancing mass transport, have also been addressed using specialized spinning disc or oscillatory photoreactors.[^103^, ^104^] Transferring synthesis from batch to continuous operation (e.g., with plug flow or (a series of) continuously stirred photoreactors) accelerates process optimization, improves handling of multiphase reaction systems, and accelerates transfer from lab to application.[^94^, ^99^, ^105^, ^106^]

**Flow photoreactors**. Over the last decade, continuously operated photomicroreactors have received widespread attention in the field of flow photochemistry.[^12^, ^82^] Their small dimensions (hundreds of micrometers to several millimeters) enable fast heat and mass transport and thus process intensification.[^107^, ^108^, ^109^] Their small size is also beneficial for homogeneous irradiation with small photon flux gradients, ensuring high reaction control for synthesis and mechanistic understanding.^[110]^ In addition, these systems are ideal models for standardized operation, and they facilitate transfer of light‐driven processes to a technical scale.[^97^, ^111^, ^112^, ^113^]

## 4 Performance Indicators in Light‐driven Catalysis: Benefits and Limitations

### 4.1 General Considerations

Accurate determining and reporting of experimental parameters for light‐driven systems is the basis for performance comparison and provides critical information for identifying future directions in the field (Figure 3). However, photochemistry is a diverse research field, and each sub‐area uses a distinct set of performance indicators which focus on specific aspects of photochemical performance. This restricts comparability and limits further development in the field.[^24^, ^25^] Thus, the authors see an urgent need for standardized reporting of performance parameters that fully describe the system studied (Figure 3). While challenging, the authors believe that this would be an enormous boost for light‐driven catalysis research, as—for the first time—comparability across different labs and even across diverse research communities based on a set of unbiased experimental criteria would become possible.

Note, however, that comparison between reaction systems is not always meaningful, even if all relevant parameters have been reported. It also requires in‐depth understanding of the reaction systems. For example, using photon flux as a basis for comparison is only meaningful if the systems to be compared are all limited by photon flux. This is often not the case; e.g., in photo‐initiated radical reactions or systems where light‐harvesting units are coupled with additional catalysts. Thus, meaningful comparison requires both comprehensive parameter reporting and understanding of the underlying reaction processes.

### 4.2 Performance Indicators Normalized to Amounts

The absolute molar amount of a target product formed is the basis of any catalyst evaluation and should be reported to facilitate data evaluation and comparison; however, absolute molar amounts are typically normalized with respect to other parameters, such as the amount of light absorber or catalyst, or the reaction time.

**Figure 4:**
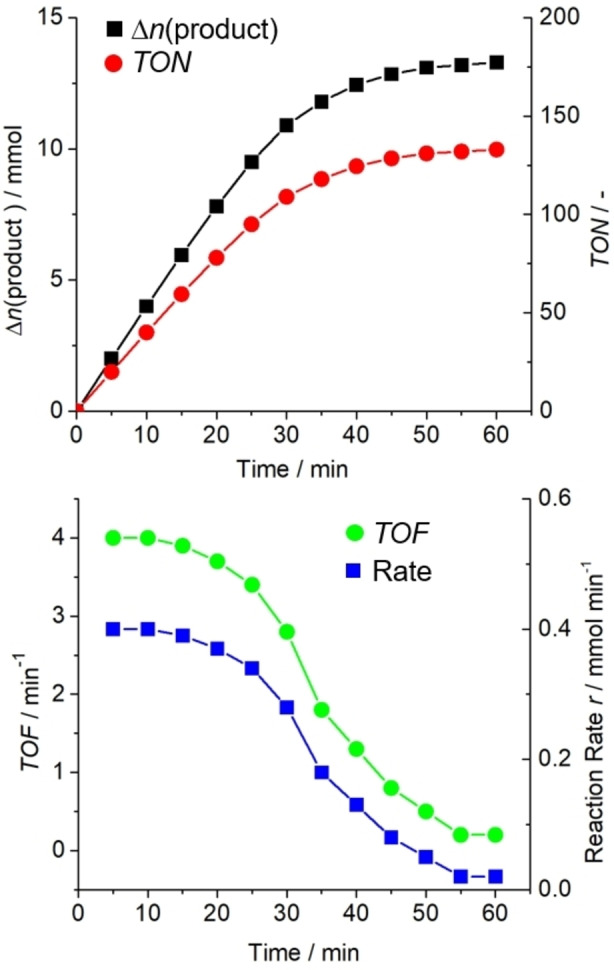
Example graphs illustrating the relationship between Δ*n*(product), *TON*, *TOF*, and reaction rate *r*.

The turnover number (*TON*) is one classical example for this approach, where the amount of product formed (or, less commonly, the amount of reactant converted) is normalized to the amount of a catalytically active component, resulting in the following definition 1 (also see Figure 4):

(1) $$TON=\frac{\Delta n\left(product\right)}{n\left(catalytically\;active\;species\right)}$$

Note that this seemingly simple equation has sparked major debates in the context of comparing catalytic performances, partly based on the following considerations:^[114]^ determining the change of the amount of product Δ*n*(product), which is often equal to *n*(product formed), can be affected by delayed product release; e.g., from heterogeneous porous catalysts^[115]^ or, for gaseous products, by slow diffusion from the liquid to the gas phase.^[40]^

The determination of *n*(catalytically active species) can be challenging if the number of sites that are accessible and active are not known (e.g., in heterogeneous catalysts).^[114]^ Thus, *n*(light‐absorbing species) is sometimes used instead of *n*(catalytically active species); e.g., when photosensitizer degradation limits the catalytic performance.

If the molar amount of an active species is unknown, the total mass *m* of an active material can be used as alternative for normalization, giving *TON_m_*:

(2) $$TON_{m}=\frac{\Delta n\left(product\right)}{m\left(active\;material\right)}$$

While rather common in thermal catalysis, this approach is not meaningful for light‐driven reactions since the correlation between activity and mass of catalytically active species is not always linear.^[116]^ We therefore recommend avoiding *TON_m_* as a performance indicator in light‐driven reactions.

### 4.3 Performance Indicators Normalized to Time

Normalization of converted or produced molar amounts with respect to the reaction time gives a reaction rate *r*, which can be calculated for a given reaction period, or averaged over the full reaction time:

(3) $$r=\frac{\Delta n\left(product\right)}{\Delta t}$$

Note that average reaction rates have only limited significance, as they can vary significantly as the reaction progresses. Reporting the reaction rates as a function of the reaction time is recommended to provide an in‐depth understanding of their changes over time (Figure 4).

The turnover frequency (*TOF*) is a commonly used parameter that reflects changes of *TON* with reaction time:

(4) $$TOF=\frac{d\;TON}{d\;t}$$

Note that the limitations described for *TONs* also apply for *TOFs*; moreover, for the reasons described above for *r*, *TOFs* averaged over the full reaction time provide limited insights only.

Simultaneous normalization of the converted or formed amount to the reaction time and reaction volume results in the space–time yield (*STY*):

(5) $$STY=\frac{\Delta n\left(product\right)}{\Delta tV\left(reaction\;solution\right)}$$

The *STY* can be calculated without any knowledge about the catalyst, making it a useful parameter when no or little information about the number of catalytically active sites is available.

### 4.4 Performance Indicators Normalized to Photons

**Figure 5:**
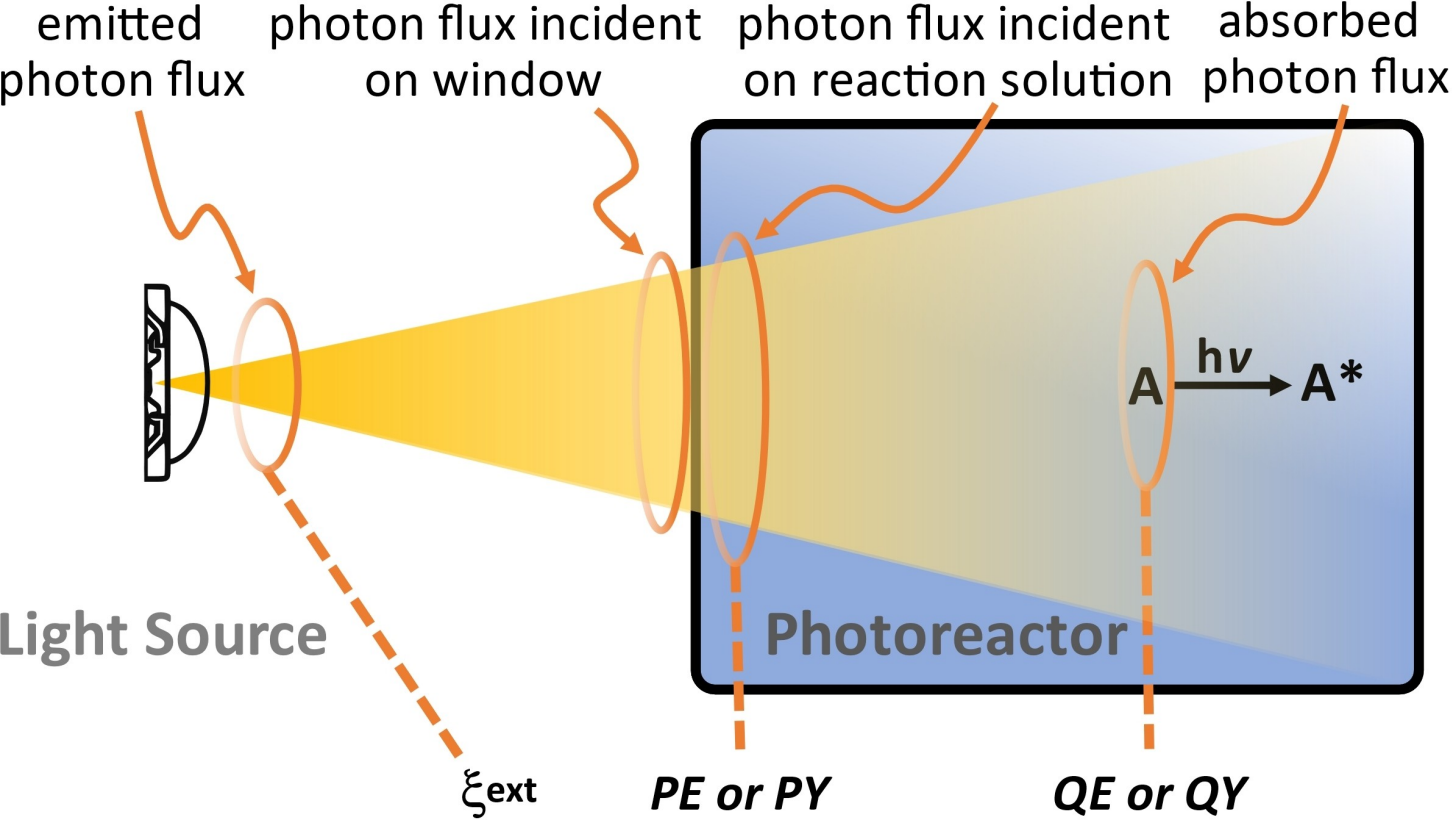
Photon‐based performance indicators and their relation to the photochemical experimental setup; light‐absorbing species (A), photonic efficiency (PE), photonic yield (PY), quantum efficiency (QE), quantum yield (QY).[^72^, ^73^]

If catalytic processes are limited by the photon flux, then normalization to photons enables calculation of the photonic efficiency *PE* (also see Figure 5):

(6) $$PE=\frac{\Delta n\left(product\right)}{\Delta n\left(incident\;photons\right)}=\frac{r}{incident\;photon\;flux}$$

Note that several similar definitions of photon‐based performance indicators exist, which vary depending on the “type” of photon considered (e.g., monochromatic, polychromatic, incident, or absorbed).[^72^, ^73^] In general, the term “yield” refers to monochromatic excitation, while the term “efficiency” refers to excitation with photons of a defined wavelength interval (i.e., polychromatic irradiation). The term “quantum” considers photons that were *absorbed* by the system, while the term “photonic” describes *incident* photons; i.e., photons that are available inside the reactor.

The external photonic efficiency ($\xi_{ext}$ ) is a variation of *PE* and can be used to evaluate the efficiency of the experimental setup rather than the efficiency of the catalyst^[117]^ (see also Figure 5):

(7) $$\xi_{ext}=\frac{r}{emitted\;photon\;flux}$$

When sunlight‐ or solar‐simulator‐driven chemical reactions are studied, the solar‐to‐chemical conversion efficiency (*SCE*) can be calculated using the Gibbs free energy change Δ*G*:

(8) $$SCE=\frac{r\Delta G}{incident\;radiation\;power}$$

*SCE* is based on well‐defined irradiation conditions (intensity and spectrum); however, it can only be used for systems that operate with primarily visible light. In contrast to *PE*, *SCE* provides no information on the spectral response of the system.

## 5 Data Management

### 5.1 Data Recording, Documenting, and Reporting

Performance indicators are prone to systematic errors caused by the experimental setup. Given the broad range of parameters that affect light‐driven catalytic activity, the authors see an urgent need for a detailed and consistent recording, documenting, and reporting of experimental data to enable independent evaluation and comparability, as well as reproduction of catalytic performance.[^24^, ^25^] This includes common (photo)chemical details but also requires careful documentation of technical information. To date, details of the irradiation conditions or exact reactor setup are often not described sufficiently, which can complicate or even inhibit comparison. Reporting the sensitivity of a reaction towards changes of certain parameters might be an initial step towards reproducibility.^[23]^ However, to identify future research directions and critical limitations in current light‐driven catalysis, the community must accept the need for, and implement, detailed reporting of the chemical and technical conditions of any given experiment. Complete data reporting, if possible in machine readable files, may form the basis for automated data management and analysis of Big Data as a means to identify reactivity trends and research directions; e.g., using machine learning[^32^, ^118^] or correlation analyses.^[119]^

### 5.2 Future Developments and Open Data Publication

To date, there are no standardized reporting strategies that describe a minimum experimental dataset required for publication of catalytic research results.[^24^, ^25^, ^120^] The advent of Big Data provides powerful tools to handle huge data sets and thus removes the limitation of only reporting integral performance indicators. Results from online and/or in situ analytics with spatially and temporally resolved information can easily be included as input for data science methods such as machine learning and multivariate correlation analysis.[^32^, ^120^] Use of the four FAIR principles—Findability, Accessibility, Interoperability, and Reusability—is a necessary step towards Big Data analysis.^[31]^ This promising approach clearly relies on the public accessibility of machine‐readable, reliable, and complete standardized datasets. Research consortia worldwide are currently developing database infrastructure to facilitate this approach by providing structured data.[^121^, ^122^] Note that peer‐review and editorial processes can play major roles to ensure that all relevant experimental data are made available along with the scientific publication.

Thus, a future database for light‐driven catalysis requires a consensus on the specifications for reported data and information. This is a crucial task for the communities involved and will have major long‐term impacts on the development of light‐driven chemistry.[^121^, ^122^] Pioneering work on national and international open data frameworks are currently underway; e.g., within the European EUDAT Collaborative Data Infrastructure initiative or the German National Research Data Infrastructure NFDI framework.^[121]^

### 5.3 Automated Data Generation by High‐Throughput Experimentation

The complexity of light‐driven catalysis will still require experimental studies to assess and confirm predictions from simulations and provide experimental evidence. Here, high‐throughput experimentation[^123^, ^124^] can become a go‐to approach for fast, reproducible generation of standardized catalysis datasets. In addition, concepts for autonomous reaction discovery are currently being developed and could become available for catalysis research.[^125^, ^126^, ^127^, ^128^] In this context, the use of continuous reactors together with online and in situ analytics will be essential for implementing these approaches in catalytic laboratories.^[12]^ A direct link between high‐throughput experiments to databases will then allow the large number of data recorded to be harnessed, enabling comprehensive evaluation, comparison, and correlation. As an additional tool, statistical design of experiment (DoE) methods can be employed for the fast and reliable identification of optimum performance conditions.^[129]^

## 6 Suggestions for a Minimum Set of Data to be Reported in Light‐Driven Catalysis

Performance indicators are only meaningful when reported together with a comprehensive set of experimental details. The authors envision that the research community will commit to the advantages of standardized reporting and Big Data and will move to publishing strategies that give access to fundamental experimental data, such as amounts and component concentrations as a function of time. This would allow independent and retrospective calculation of performance indicators.

Table 1 proposes a pragmatic minimum set of data to be reported in light‐driven catalysis. The table is based on many discussions of the authors with colleagues from the photochemical community. Note that this table forms the starting point for a community‐driven definition of standard data to be reported in light‐driven catalysis.

**Table 1:** First version of the suggested dataset to enable objective comparison of light‐driven catalytic experiments.

Type of Parameters	Parameter	Comments
**Chemical Parameters**	purity	for all chemical species used
	concentrations and molar amounts	for all chemical species used
	pH value/proton concentration	
	absorption coefficients as function of wavelength	of all light‐absorbing species
**Reactor**	materials	
	geometry	
	dimensions	
**Experimental Setup**	positioning	reactor, light source, filter
**Operation Conditions**	reaction volume	
	temperature	ideally time resolved
	atmosphere and pressure	pressure ideally time resolved
	operation mode	continuous, batch, semi‐continuous
	stirring speed	if applicable
	flow rates	if applicable
	reaction time	
**Light Sources**	emission spectrum	
	photon flux	ideally within the reactor, alternatively, at the outside reactor wall
**Performance Indicators**	*n*(product)	ideally time resolved
	*TON*	molecular systems; ideally time resolved
	*TOF*	molecular systems; time resolved
	reaction rate	heterogeneous systems; time resolved
	*STY*	
	$\xi_{ext}$	
	*SCE*	for systems driven by solar irradiation

To achieve this goal, this table is provided as an open document in a persistent repository (https://github.com/photonZfeed/photoComparison).^[130]^ All readers are encouraged to contribute to the development of the parameter table by providing constructive suggestions for changes to the table, so that over time, the table can be adapted to the requirements of the community and to new developments in the field. Curated versions of the table will regularly be uploaded as up‐to‐date, versions‐of‐record, which can be accessed and cited via a persistent digital object identifier (https://doi.org/10.5281/zenodo.5911870).^[131]^ This approach will ensure full transparency in terms of contents of the table, and the use of open contributor repositories will allow the community to actively engage in documented discussions on the contents of the list, and maybe even broader questions in research data comparability. The authors kindly ask the community to support this endeavor by sharing their suggestions for development of the list via the provided repository link.

## 7 Conclusion

Light‐driven catalysis is a thriving field with major importance for energy technologies, sustainability, and green industrial processes. The identification of the most promising high‐performance systems for a given task is currently challenging, as the collection, recording, and reporting of critical performance indicators is not unified across the research communities. To date, unbiased evaluation and comparison between systems is therefore nearly impossible. This Scientific Perspective highlights the current challenges in determining meaningful comparability data based on an analysis of critical system variables, key performance indicators are identified, and their benefits and limitations are discussed together with a suggestion of the minimum set of data that should be reported in publications concerning light‐driven catalysis. In addition, recent trends in the application of statistical and computational methods are highlighted that could cope with the large amount of data and complexity of typical light‐driven catalytic systems. When combined with the rapidly evolving capabilities in data science and ever‐increasing computational power, these methods can utilize reported experimental data to accelerate the rational design and knowledge‐based optimization of light‐driven catalytically active systems.

## Conflict of interest

The authors declare no conflict of interest.

8

## Biographical Information

*Dirk Ziegenbalg is Professor of Chemical Engineering at Ulm University. His current research deals with photochemical reaction engineering and design of photoreactors at the interface between chemical engineering, microreaction technology, and photochemistry, aiming for highly efficient photochemical processes*.

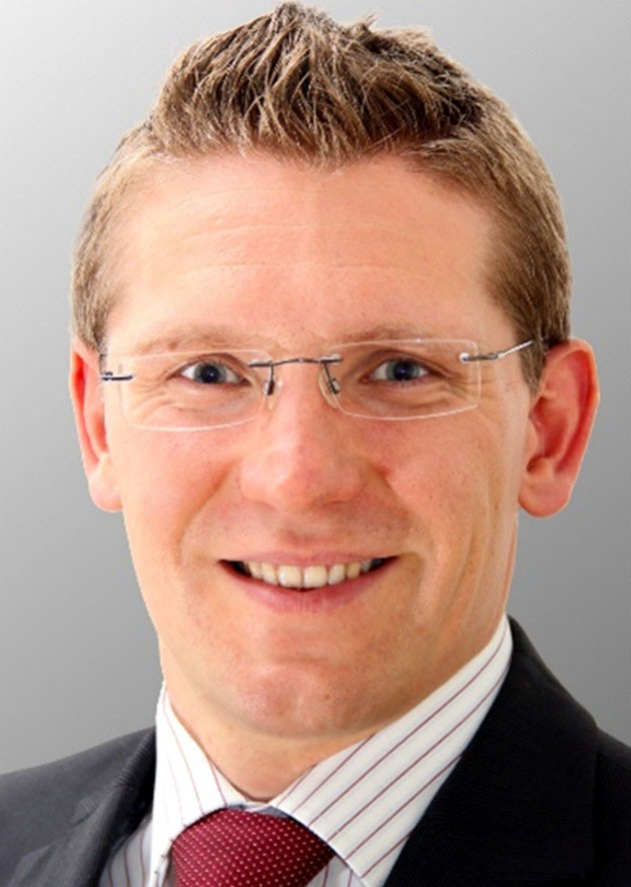

## Biographical Information

*Andrea Pannwitz is Junior Professor of Inorganic Chemistry and Energy Conversion at Ulm University. Her current research focuses on photocatalysis and energy transfer within self‐assembled lipid bilayer membranes and compartmentalizing vesicles using active molecular components*.

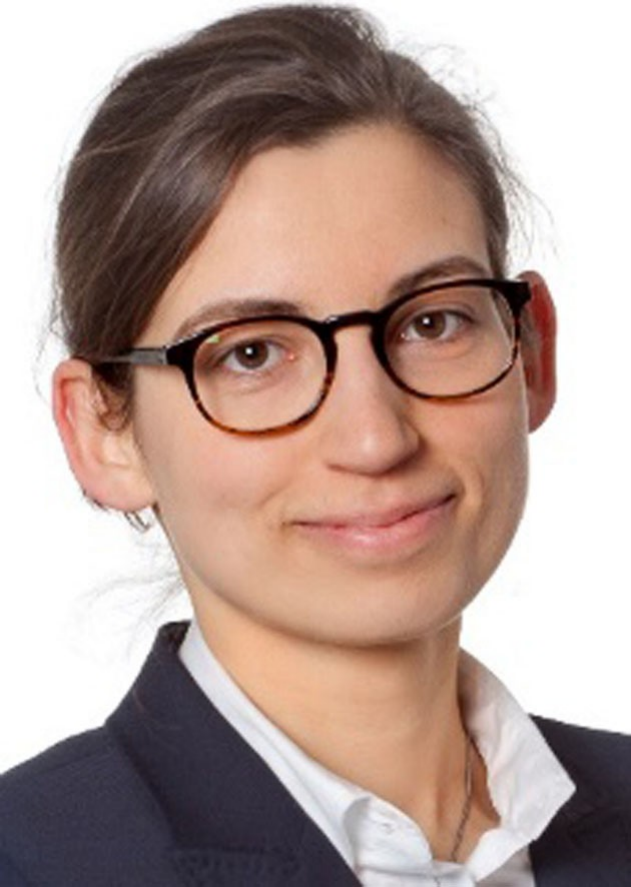

## Biographical Information

*Sven Rau is Professor of Inorganic Chemistry at Ulm University, and spokesperson of the Collaborative Research Center TRR 234 “CataLight”. His research interests are the design of photochemical (supra)molecular devices based on metal complexes for light‐driven hydrogen evolution, water oxidation, and other energy‐relevant chemical conversions*.

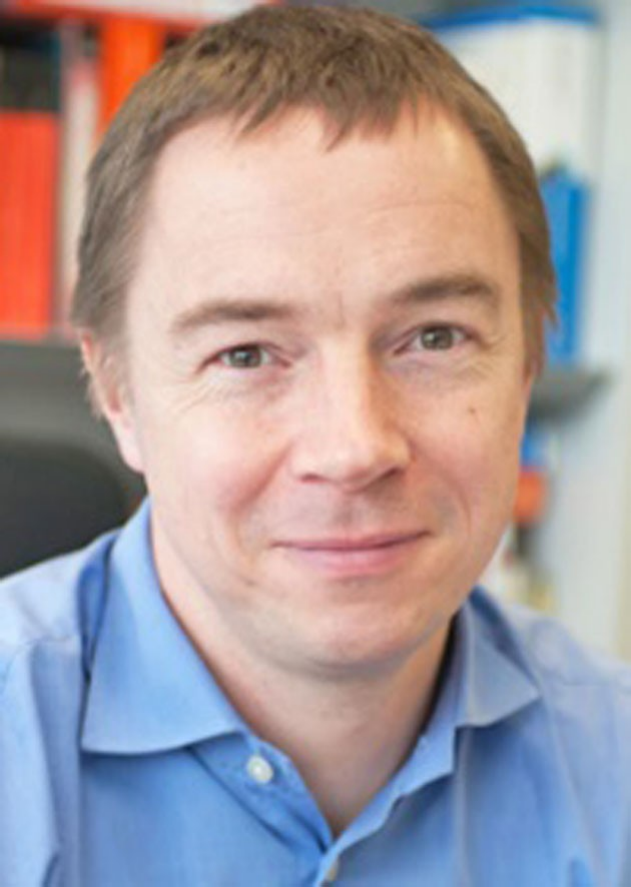

## Biographical Information

*Benjamin Dietzek‐Ivanšić is Professor of Physical Chemistry at Friedrich Schiller University and heads the research department Functional Interfaces at the Leibniz Institute of Photonic Technology. He is interested in studying photoactive molecules and materials; e.g., in photocatalysis*.

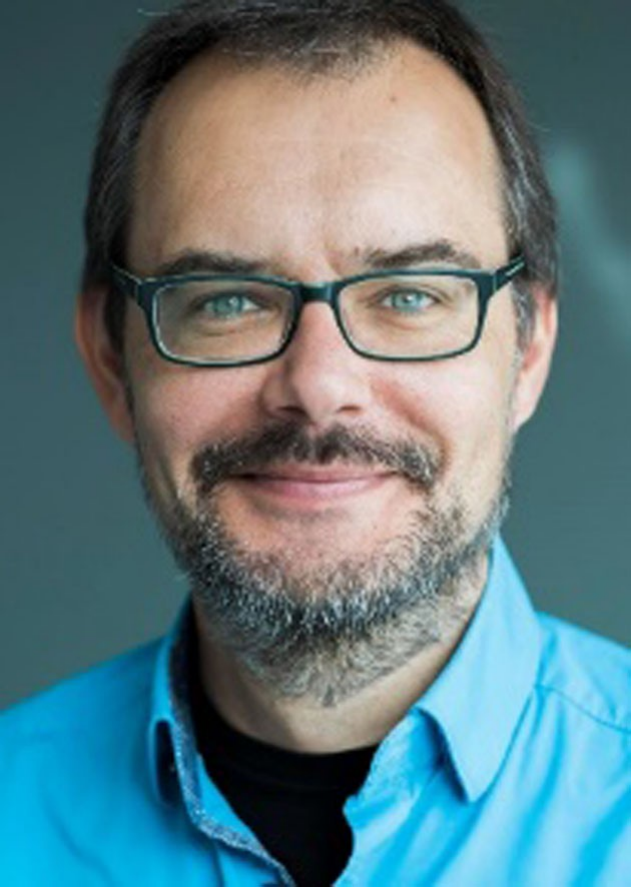

## Biographical Information

*Carsten Streb is currently Professor of Inorganic Chemistry at Johannes Gutenberg University Mainz. His current research is focused on designing polyoxometalate‐based functional materials and composites to address global chemical challenges with a focus on energy conversion and energy storage*.

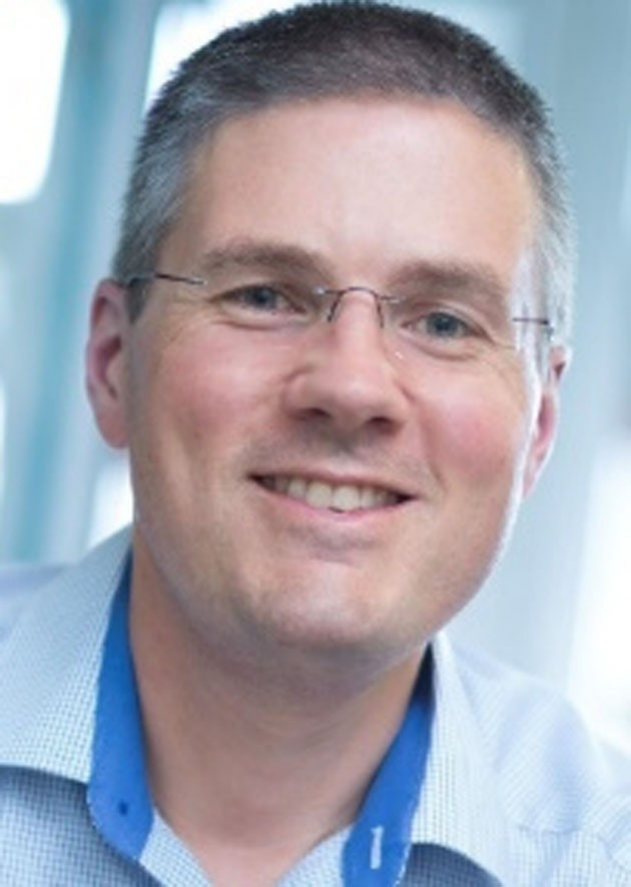

## Supporting information

As a service to our authors and readers, this journal provides supporting information supplied by the authors. Such materials are peer reviewed and may be re‐organized for online delivery, but are not copy‐edited or typeset. Technical support issues arising from supporting information (other than missing files) should be addressed to the authors.

Supporting Information

## Data Availability

The data that support the findings of this study are openly available on Zenodo.org at https://doi.org/10.5281/zenodo.5911869 (Concept DOI) or at https://doi.org/10.5281/zenodo.5911870 (Version 1.0.0 DOI). A community‐driven definition of standard data for reporting in light‐driven catalysis is collated in a repository on GitHub.com at https://github.com/photonZfeed/photoComparison; curated versions of this data will be uploaded to Zenodo.org on a regular basis and should be cited using the relevant Version DOI or, when citing the evolving research artifact, the Concept DOI. The Publisher cannot be held responsible for the contents and contemporaneity of any external websites.
