# Supplementary material for: Comparative Evaluation of Light‐Driven Catalysis: A Framework for Standardized Reporting of Data
Source: Angew Chem Int Ed Engl. 2022 Jun 13;61(28):e202114106. doi: 10.1002/anie.202114106 (PMC9401044; doi:10.1002/anie.202114106)
Supplement: Supplementary file 1 — Supporting Information [file ANIE-61-0-s001.pdf]

Angewandte Chemie
Eine Zeitschrift der Gesellschaft Deutscher Chemiker

Supporting Information

Comparative Evaluation of Light-Driven Catalysis: A Framework for Standardized Reporting of Data

D. Ziegenbalg, A. Pannwitz, S. Rau, B. Dietzek-Ivanšić, C. Streb**

Supporting Information

- example-worksheet_empty_v3.xlsx
- example-worksheet_v3.xlsx
